# Class 1 Integrons in Resistant *Escherichia coli* and *Klebsiella* spp., US Hospitals

**DOI:** 10.3201/eid1206.051596

**Published:** 2006-06

**Authors:** Aarati N. Rao, Miriam Barlow, Leigh Ann Clark, John R. Boring, Fred C. Tenover, John E. McGowan

**Affiliations:** * Emory University, Atlanta, Georgia, USA;; † Centers for Disease Control and Prevention, Atlanta, Georgia, USA

**Keywords:** antimicrobial resistance, integron, Escherichia coli, Klebsiella, surveillance

## Abstract

We examined *Escherichia coli* and *Klebsiella* spp. from US hospitals for class 1 integrons. Of 320 isolates, 181 (57%) were positive; association of integrons with resistance varied by drug and organism. Thus, determining integron epidemiology will improve understanding of how antibacterial resistance determinants spread in the United States.

Integrons are genetic elements, located on the bacterial chromosome or a plasmid, that often carry genetic determinants for antimicrobial drug resistance (*1*). The need for systematic epidemiologic studies of the role of integrons in antimicrobial drug resistance in bacteria has recently been emphasized (*2*). The prevalence of integrons is high among gram-negative isolates from patients in Europe (*3**,**4*), and some carry multiple integrons (*3*). Reports from Asian countries also have noted a high prevalence of class I integrons in gram-negative clinical isolates (*5*). Most of the resistance integrons found to date in clinical isolates of *Enterobacteriaceae* are class 1 integrons, which are highly associated with resistance to antimicrobial agents (*2*).

These data suggest that integrons are relatively common, especially among the *Enterobacteriaceae*, and that they contribute to the spread of antimicrobial drug resistance in healthcare settings. However, few studies from the United States have assessed the association between integron carriage and antimicrobial susceptibility patterns.

This study analyzes the association between class 1 integrons and resistance to selected antimicrobial agents in a convenience sample of *Escherichia coli* and *Klebsiella* isolates. Multivariate analysis was used to determine whether apparent associations were affected by interactions between variables. Isolates were submitted from hospitals participating in Project ICARE (Intensive Care Antimicrobial Resistance Epidemiology) (*6*). The protocol has been approved as exempt by the institutional review board at Emory University.

## The Study

Clinical isolates of *E. coli*, *Klebsiella pneumoniae*, and *K. oxytoca* were obtained from 19 US hospitals during phase IV (2002–2004) of Project ICARE, which focused on nonoutbreak isolates of *Enterobacteriaceae* with decreased susceptibility to extended-spectrum cephalosporins, fluoroquinolones, or carbapenems. The 19 hospitals were located in 13 states throughout the country and had an approximately equal mix of teaching and nonteaching medical centers. Duplicate isolates from the same patient were excluded.

Isolates were tested for susceptibility to the antimicrobial drugs listed in Table 1 by using the broth microdilution reference method (BMD) described by the Clinical and Laboratory Standards Institute (*7*). Isolates were stored at –70°C and were subcultured to trypticase soy agar plates containing 5% defibrinated sheep blood (BD BioSciences, Sparks, MD, USA) >2 times before testing. For each organism, BMD tests were inoculated by using a cell suspension equivalent to a 0.5 McFarland standard. *Enterobacteriaceae* isolate identifications provided by participating laboratories were confirmed by colony shape, spot tests (*8*), and Vitek GNI+ cards (bioMérieux, Durham, NC, USA). Differences were resolved by using reference biochemical tests (*9*).

**Table 1:** Crude association between nonsusceptibility to various antimicrobial drugs and positive test result for class 1 integron in *Escherichia coli* and *Klebsiella* spp. (*Klebsiella pneumoniae* and *K. oxytoca*)

Class/agent	*E. coli* (n = 209), OR (95% CI)*	*Klebsiella* spp. (n = 111), OR (95% CI)*
Aminoglycosides		
Amikacin	3.15 (0.32–30.79)	4.0 (0.86–18.51)
Gentamicin	**5.04 (2.69–9.47)**	**4.49 (1.80–11.22)**
Tobramycin	**5.55 (3.01–10.23)**	**5.0 (2.0–12.52)**
Fluoroquinolone		
Ciprofloxacin	**3.00 (1.40–6.42)**	1.33 (0.59–3.04)
3rd-generation cephalosporin		
Cefotaxime	1.14 (0.63–2.07)	2.26 (0.99–5.19)
Cefpodoxime	1.02 (0.59–1.75)	**2.61 (1.05–6.52)**
Ceftazidime	1.30 (0.73–2.31)	**2.54 (1.10–5.88)**
4th-generation cephalosporin		
Cefepime	0.35 (0.11–1.13)	1.87 (0.57–6.10)
Monobactam		
Aztreonam	1.58 (0.87–2.89)	1.10 (0.48–2.53)
β-Lactamase inhibitor		
Piperacillin-tazobactam	1.40 (0.75–2.62)	1.82 (0.80–4.14)
Others		
Chloramphenicol	**2.33 (1.30–4.17)**	2.29 (0.97–5.42)
Minocycline	1.57 (0.90–2.76)	1.20 (0.53–2.71)
Trimethoprim-sulfamethoxazole	**12.24 (6.28–23.86)**	**3.68 (1.57–8.62)**

*OR, odds ratio; CI, confidence interval. Variables with p values £0.05, in **boldface**, are considered significant.

Isolates were analyzed by polymerase chain reaction (PCR) amplification techniques to determine whether a class 1 integron was present. Integrons were detected by PCR amplification of a class 1 integrase–specific fragment of the *IntI1* gene as previously described (*1*). The primer sequences used were IntI1-F: 5´-TCT CGG GTA ACA TCA AGG-3´ and IntI1-R: 5´-AGG AGA TCC GAA GAC CTC-3´.

Amplifications were performed in 10 μL of Taq PCR Master Mix (Qiagen, Valencia, CA, USA), 1.5 mmol/L MgCl_2_, 5 pmol/L each primer, and 1 μg template DNA (*1*). DNA was extracted with a Genelite Bacterial Genomic DNA Kit (Sigma, Saint Louis, MO, USA). Amplification specifications were as follows: 5 min at 94°C followed by 35 cycles of 1 min at 94°C, 1 min at 55°C, and 30 s at 72°C. PCR products were analyzed by gel electrophoresis with 1.5% agarose gels. All PCRs included positive and negative controls.

A statistical comparison of the frequencies of integron presence in *E*. *coli* and *Klebsiella* spp. was conducted by using odds ratios and 95% confidence intervals. Intermediate and resistant isolates were pooled as nonsusceptible for analysis. Multivariate logistic regression analysis was conducted with 1 representative from each major class of drugs tested. Data validation and analysis were performed by SAS statistical software version 9.1 (SAS Institute, Cary, NC, USA). Fit of the data to this model was evaluated by the Hosmer-Lemeshow χ^2^ test.

A positive test result for class 1 integrons was found for 181 (57%) of the 320 bacterial isolates screened, including 103 (49%) of 209 *E. coli* isolates and 78 (70%) of 111 *Klebsiella* spp. isolates. A positive test result in *E. coli* isolates was significantly associated with nonsusceptibity to gentamicin, tobramycin, ciprofloxacin, chloramphenicol, and trimethoprim-sulfamethoxazole (Table 1). A positive test result in *Klebsiella* isolates was significantly associated with nonsusceptibility to gentamicin, tobramycin, cefpodoxime, ceftazidime, and trimethoprim-sulfamethoxazole.

Multivariate analysis showed that the only drug variables associated with a positive test result for class 1 integrons in *E. coli* were nonsusceptibility to gentamicin and trimethoprim-sulfamethoxazole (with a strong interaction between the 2 variables), while an inverse association was present between a positive integron test result and nonsusceptibility to cefepime (Table 2). For *Klebsiella* spp., a positive association existed between a positive integron test result and nonsusceptibility to gentamicin and trimethoprim-sulfamethoxazole, as well as an inverse association between a positive integron test result and nonsusceptibility to aztreonam.

**Table 2:** Association of nonsusceptibility to various antimicrobial agents and presence of integrons in *Escherichia coli* and *Klebsiella* spp. (*Klebsiella pneumoniae* and *K. oxytoca*)*

Antimicrobial drug	Estimate	OR (95% CI)	p value†
*E. coli*			
Gentamicin‡	1.16	3.19 (1.40–7.30)	0.006
Ciprofloxacin	0.31	1.37 (0.46–4.03)	0.573
Ceftazidime	–0.81	0.45 (0.14–1.45)	0.180
Cefepime	–1.77	0.17 (0.04–0.82)	0.027
Piperacillin-tazobactam	–0.38	0.68 (0.28–1.66)	0.407
Aztreonam	1.19	3.29 (0.90–12.0)	0.071
Chloramphenicol	0.29	1.34 (0.60–2.99)	0.476
Minocycline	–0.11	0.89 (0.39–2.06)	0.791
Trimethoprim-sulfamethoxazole‡	2.56	12.9 (5.73–29.0)	<0.001
Source§	1.10	3.02 (1.23–7.45)	0.02
Intercept	–2.43		<0.0001
*Klebsiella* spp.			
Gentamicin	1.44	4.23 (1.48–12.03)	0.007
Ciprofloxacin	0.34	1.40 (0.43–4.56)	0.578
Ceftazidime	0.92	2.51 (0.49–12.80)	0.270
Cefepime	0.05	1.05 (0.27–4.14)	0.942
Piperacillin-tazobactam	0.02	1.02 (0.26–4.07)	0.979
Aztreonam	–1.64	0.19 (0.04–0.99)	0.048
Chloramphenicol	–0.57	0.57 (0.13–2.44)	0.447
Minocycline	0.13	1.14 (0.35–3.73)	0.835
Trimethoprim-sulfamethoxazole	1.52	4.61 (1.39–15.29)	0.012
Source§	0.56	1.75 (0.64–4.78)	0.275
Intercept	–0.28		0.591

*Multivariate logistic regression with variables included for 1 drug of each major class that was tested. Hosmer and Lemeshow goodness-of-fit test statistic: *E. coli* total χ^2^ = 10.852, *df* = 8, p = 0.21; *Klebsiella* spp. total χ^2^ = 4.538, *df* = 8, and p = 0.81. OR, odds ratio; CI, confidence interval. †By χ^2^ approximation. ‡Significant interaction between these 2 variables (p<0.001). §Nonurine source vs. urine source (reference).

## Conclusions

More than half of the selected isolates from US hospitals that we tested were positive for class 1 integrons. The prevalence of class 1 integrons in *E. coli* was 49% in selected non-outbreak isolates from hospitalized patients from 2002 to 2004 in our study, 52% in consecutive urinary tract isolates in 2001 in Sweden (*10*), and 15% in isolates from blood in hospitals in Sweden in 1998 and 1999 (*11*). The prevalence of integrons was 70% in *Klebsiella* isolates from hospitalized patients in our study and 73% in extended-spectrum β-lactamase (ESBL)–producing *Klebsiella* spp. collected from 3 hospitals in Australia from 1989 to 1999 (*12*). These data cannot be directly compared because of differences in selection criteria and testing procedures, but as a whole they suggest that prevalence of integrons in these bacteria in the United States is high, as it is in other areas of the world. Carbapenem resistance was infrequent in the isolates we tested, so we could not address the association of integron carriage and metallo-β-lactamases that has been observed in other parts of the world (*2*).

Our study found an inverse association between integron presence and nonsusceptibility to cefepime in *E. coli* and to aztreonam in *Klebsiella* spp., which indicates that resistance determinants for these drugs are not frequently carried by integrons. One possible reason for the lack of an association between nonsusceptibility to cefepime and integron carriage is the relatively recent approval of cefepime for use in the United States. CTX-M-type β-lactamases are the most common resistance determinants in cefepime-resistant isolates worldwide, but these enzymes are just beginning to appear in the United States (*13*).

In phase IV of Project ICARE, participating sites were asked to submit isolates that showed decreased susceptibility to fluoroquinolones, extended-spectrum cephalosporins, or carbapenems. We did not track whether isolates were from patients in intensive care units or not. Thus, our isolates made up a convenience sample strongly associated with drug resistance mechanisms carried as integron gene cassettes and otherwise, and they are not necessarily representative of isolates from the United States in general. We did not examine carriage of >1 integron, as documented in earlier studies (*10*).

In these selected isolates, the crude association between a positive test result for class 1 integrons and nonsusceptibility to a fluoroquinolone (ciprofloxacin) in *E. coli*, and nonsusceptibility to a β-lactam (ceftazidime) in *Klebsiella* spp. failed to remain as independent variables in the multivariate model. This finding suggests that the mechanisms of resistance to fluoroquinolones and β-lactams are associated with mechanisms of resistance to the drugs that remained independently associated with class 1 integrons (aminoglycosides and trimethoprim-sulfamethoxazole), but for reasons unrelated to integron carriage. A previous study also found that ciprofloxacin resistance no longer predicted integron presence in *Enterobacteriaceae* when multivariate analysis was conducted (*1*). A Spanish study (*14*) found no association between integron carriage and β-lactam resistance in ESBL-producing *E. coli* strains unless strains contained metallo-β-lactamases, which were infrequently encountered in our study population. A biologic mechanism for association of resistance to fluoroquinolones and aminoglycosides (i.e., the inactivation of ciprofloxacin by the *aac* (*6´*)-*Ib*-*cr* aminoglycoside acetylating gene) has been recently reported (*15*).

In our study, both crude and multivariate associations between integron positivity and nonsusceptibility varied for the *E. coli* and *Klebsiella* groups. This finding emphasizes that studies of this type must assess whether analysis should combine results for organisms of different genus and species into larger groups (e.g., *Enterobacteriaceae*).

When determinants encoding resistance to a variety of antimicrobial classes are contained within an integron, use of any of these agents may select for and enhance expression of the other determinants (*2*). Thus, in the example of our studied isolates, use of aminoglycosides may lead to spread of a multidrug-resistant bacterial strain such as *K. pneumoniae*. The differential association of class 1 integrons with resistance in the isolates we studied suggests that integrons facilitate the spread of antimicrobial drug resistance in the United States.
